# Techno-economic analysis of sidestream ammonia removal technologies: Biological options versus thermal stripping

**DOI:** 10.1016/j.ese.2022.100220

**Published:** 2022-11-08

**Authors:** Pascal Ochs, Ben Martin, Eve Germain-Cripps, Tom Stephenson, Mark van Loosdrecht, Ana Soares

**Affiliations:** a Cranfield University, College Road, Cranfield, Bedford, MK43 0AL, United Kingdom; b Thames Water, Reading STW, Island Road, RG2 0RP, Reading, United Kingdom; c Delft University of Technology, Building 58, Van der Maasweg 9, 2629, Delft, Netherlands

**Keywords:** Deammonification, Nitrification, Denitrification, Wastewater, Sludge dewatering liquors

## Abstract

Over the past twenty years, various commercial technologies have been deployed to remove ammonia (NH_4_–N) from anaerobic digestion (AD) liquors. In recent years many anaerobic digesters have been upgraded to include a pre-treatment, such as the thermal hydrolysis process (THP), to produce more biogas, increasing NH_4_–N concentrations in the liquors are costly to treat. This study provides a comparative techno-economic assessment of sidestream technologies to remove NH_4_–N from conventional AD and THP/AD dewatering liquors: a deammonification continuous stirred tank reactor (PNA), a nitrification/denitrification sequencing batch reactor (SBR) and thermal ammonia stripping process with an ammonia scrubber (STRIP). The SBR and PNA were based on full-scale data, whereas the STRIP was designed using a computational approach to achieve NH_4_–N removals of 90–95%. The PNA presented the lowest whole-life cost (WLC) over 40 years, with £7.7 M, while the STRIP had a WLC of £43.9 M. This study identified that THP dewatering liquors, and thus a higher ammonia load, can lead to a 1.5–3.0 times increase in operational expenditure with the PNA and the SBR. Furthermore, this study highlighted that deammonification is a capable and cost-effective nitrogen removal technology. Processes like the STRIP respond to current pressures faced by the water industry on ammonia recovery together with targets to reduce nitrous oxide emissions. Nevertheless, ammonia striping-based processes must further be demonstrated in WWTPs and WLC reduced to grant their wide implementation and replace existing technologies.

## Highlights

• Ammonia removal and recovery technologies from sidestream liquors are expanding.
• The costs of three technologies were compared for THP/AD and AD dewatering liquors.
• Thermal ammonia stripping plus ammonia scrubbing could recover 49 kg N year^−1^.
• THP/AD dewatering liquors led to 1.5–3 fold increased costs compared to AD.
• Deammonification was the most cost-effective technology with £0.2–0.5 kg^−1^ N.

## 1 Introduction

Over the past years, many large wastewater treatment plants (WWTPs) have upgraded their anaerobic digestion process (AD) with pre- and post-treatment technologies to increase biogas production and reduce the dry solid content of the sludge cake. One of the most implemented pre-treatment technologies is the thermal hydrolysis process (THP) [1]. In the THP, sludge is treated with steam at 150–180 °C in batch configuration, which aims to break down macromolecules and make sludge more digestible [2]. The key benefits of combined THP and AD include improved sludge rheology that enables higher organic loading rates to the AD and thus greater biogas volumes produced and an enhanced dewaterability, thus, higher dry solid content of the final sludge cake [2,3]. Additionally, THP/AD increases the ammonia (NH_4_–N) load in the dewatering liquors produced post-AD [2] by as much as 20–30% [4]. For this reason, many WWTPs have been upgraded with sidestream NH_4_–N removal technologies to reduce the returned nutrient load to the secondary nitrification process [4].

Over the last 20 years, many commercial technologies have been developed and implemented for this purpose, e.g., deammonification, nitrification and denitrification, ion exchange, air stripping, thermally driven ammonia stripping, etc. Deammonification is a two-step biological nitrogen removal process consisting of partial nitritation and anammox that convert NH_4_–N and nitrite into nitrogen gas [5,6]. Total nitrogen removal can also be achieved by combining nitrification and denitrification. Nitrification consists of a two-step reaction that takes place in well-aerated environments where NH_4_–N is first converted into nitrite and, in the second step, into nitrate [7]. During denitrification, nitrate or nitrite is converted to nitrogen gas by heterotrophic microorganisms [8]. When comparing the biological technologies above, the benefits of deammonification over nitrification/denitrification include a 60% aeration demand reduction in aeration and no need for additional chemical supplementation (e.g., alkalinity for nitrification and organic carbon for denitrification) [9]. Both deammonification and nitrification/denitrification technologies have been commonly applied to treat sludge dewatering liquors in sidestream configuration at wastewater treatment plants (WWTP) [10,11].

When looking at non-biological options, ammonia stripping is a physiochemical nitrogen removal process that uses the balance of free NH_3_, temperature and/or pH to drive out nitrogen ions from high-strength N–NH_4_ liquid streams [10,12,13]. To ensure the process efficiency, this process takes place at pH > 10, and alkaline chemical dosing is often required [8,10]. On the other side, thermal-driven NH_4_–N stripping does not require chemical dosing as nitrogen ions transfer to the gas phase is ensured by temperatures of >90 °C [13,14]. Ammonia stripping with chemical dosing has also been widely applied for ammonia removal from sludge dewatering liquors from conventional AD [10]. However, thermal-driven ammonia stripping has not yet been applied on a full-scale in WWTP, and only a handful of lab-scale and pilot studies are available [13,15]. Previous studies reported that thermal ammonia stripping processes could remove up to 96% of NH_4_–N from AD dewatering liquors [16]. It is known that at increasing temperatures from 90 to 102 °C, about 99% of the NH_4_–N was stripped out at hydraulic retention times of 3–4 h [17]. The ammonia gas is cleaned via acid adsorption (e.g., sulphuric acid) or via water scrubbing [15,17]. Other studies successfully stripped the NH_4_–N from the digestate sludge directly from conventional AD, demonstrating ammonia removal efficiency (ARE) of 50–96% [18]. Unlike deammonification and nitrification/denitrification technologies, thermal ammonia stripping technologies have not yet been applied on a full-scale to treat NH_4_–N from THP dewatering liquors. Overall, there are limited peer-reviewed studies on thermal ammonia stripping for the application of sidestream dewatering liquors. The most frequent full-scale implementations describe air stripping technologies (ammonia loads of 45,000–200,000 kg N d^−1^) that rely on chemical dosing (e.g., sodium hydroxide, lime, etc.) to raise the pH > 10 [10]. Nevertheless, ammonia stripping has another potential advantage, as these do not oxidise ammonia, there is a possibility for its recovery. Nitrogen recovery from wastewater streams is becoming increasingly of interest since the current way to produce nitrogen for fertilisers via the Haber-Bosch process is unsustainable [19]. Thermal-driven ammonia stripping combined with scrubbing produces a range of ammonia salts (e.g., ammonium hydroxide, ammonium sulphate) that can be recovered and used as fertiliser [20,21]. Additionally, thermal-driven ammonia stripping has been described as a suitable nitrogen recovery process from sidestream liquors that do not require the addition of chemicals [20,22].

To access the potential of these technological advances, it is necessary to understand their costs and how they compare to each other. Few peer-reviewed studies describe the cost of sidestream technologies to remove NH_4_–N from conventional AD, and there is no information available on THP/AD dewatering liquors treatment with much higher NH_4_–N loads. In comparing deammonification technology and nitrification/denitrification technology [23], highlighted that deammonification technologies had lower operational expenditure (OPEX). This was also quantified by Ref. [24]; who compared an identifying annual OPEX savings of up 10%. This was much lower than frequently referenced academic papers highlighting 60% savings in aeration and 100% in chemical dosing [9]. Hence there are still many unknowns on the potential NH_4_–N removal costs from standard technologies such as nitrification/denitrification compared with PNA and thermal ammonia stripping process with an ammonia scrubber (STRIP), especially under high influent loads. This study aimed at providing and comparing the whole-life costs of different sidestream technologies for NH_4_–N removal from two different scenarios: THP/AD dewatering liquors and conventional AD dewatering liquors.

## 2 Materials and methods

### 2.1 Dewatering liquor characterisation

**Fig. 1:**
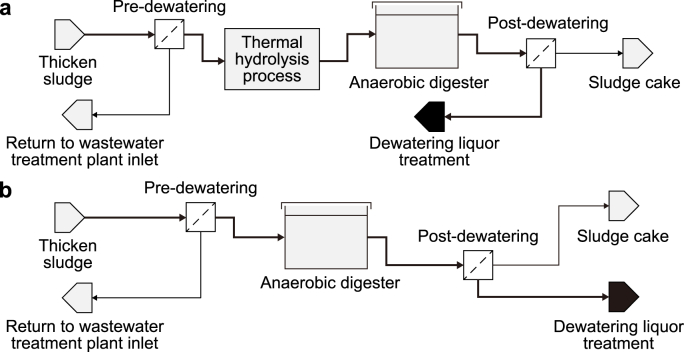
Schematic representation of Scenario 1 with dewatering liquors originating from thermal hydrolysis anaerobic digestion liquors (THP/AD) (**a**) and conventional anaerobic digestion dewatering liquors (AD) in Scenario 2 (**b**).

The influent dewatering liquors from a WWTP with 200,000 people equivalent is presented in Table 1. Scenario 1 focused on the treatment of THP/AD dewatering liquors (Fig. 1a) and had an NH_4_–N of 1700 mg N L^−1^, a soluble chemical oxygen demand (COD) of 2000 mg L^−1^, a BOD of 450 mg L^−1^, a pH of 8.5, and an alkalinity of 3000 mg CaCO_3_ L^−1^ (Table 1). Scenario 2 refers to the treatment of conventional AD dewatering liquors (Fig. 1b). The conventional AD dewatering liquor had an NH_4_–N of 750 mg N L^−1^, soluble COD of 1500 mg L^−1^, a BOD of 203 mg L^−1^, a pH of 8.0, and an alkalinity of 2500 mg CaCO_3_ L^−1^ (Table 1). The dewatering liquor temperature for both scenarios was assumed as 26 °C, according to Winter et al. [26].

**Table 1:** Characteristics for dewatering liquors from Scenario 1 THP/AD and Scenario 2 AD (Winter et al., 2017; [25].

Characteristic	Scenario 1: Thermal hydrolysis/anaerobic digestion dewatering liquors (THP/AD)	Scenario 2: Conventional anaerobic digestion dewatering liquors (AD)
Ammonia (mg N L^−1^)	1700	750
Total COD (mg L^−1^)	6700	4846
Soluble COD (mg L^−1^)	2000	1500
BOD (mg L^−1^)	450	203
Alkalinity (mg L^−1^)	3000	2500
pH	8.5	8.0

### 2.2 Process design

**Fig. 2:**
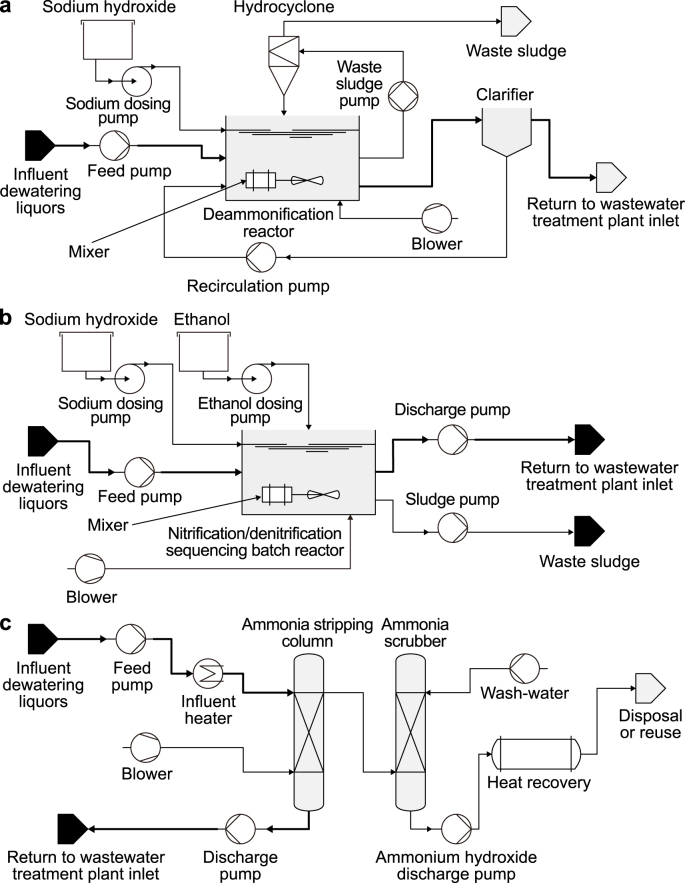
Schematic representation of suspended sludge deammonification technology (PNA) (**a**), nitrification/denitrification sequencing batch reactor (**b**), and thermal ammonia stripping with ammonia scrubbing (**c**).

Three different sidestream ammonia removal technologies were designed for Scenario 1 and Scenario 2 (Fig. 2). All evaluated technologies were designed for the same influent characteristics at a dewatering liquor flow rate of 1000 m^3^ d^−1^ (Table 1) at a wastewater treatment plant of 250,000 people equivalent similar to Ochs et al*.* (2020). The design was completed so that all technologies reached the desired effluent of 70 mg N L^−1^ NH_4_–N, 200 mg N L^−1^ nitrate, and 10 mg N L^−1^ nitrite, in agreement with full-scale [27].

The deammonification continuous stirred tank reactor (PNA) was designed based on the computational approach from Ref. [8]; the reaction stoichiometry by Ref. [6] and the microbial growth and kinetics from Ref. [28] (Fig. 2a). A design mixed liquors suspended solids (MLSS) of 3000 mg L^−1^ and dissolved oxygen (DO) concentration of 0.3 mg L^−1^ was selected. The designed PNA had a tank depth of 6 m. The solids retention time (SRT) for AMX and AOB was assumed to be separated due to the action of an hydrocyclone, as suggested in previous studies [27,28]. The SRT for AMX was 35 days [28]. No additional carbon was added to the PNA, and it was assumed that the nitrogen removal was mainly via the deammonification chain of reactions.

The nitrification/denitrification sequencing batch reactor (SBR) was designed based on the computational approach SRT described in Ref. [8] (Fig. 2c). The SBR a total cycle time was 6 h with 4.5 h and react, 1 h for settling, and 0.5 h decant. The design DO for the SBR was 2.0 mg L^−1^. The reaction period was separated into aerobic (66%) and anoxic (34%) phases for nitrification and denitrification, respectively. The SBR was dosed with sodium hydroxide to balance pH/CO_2_ and support alkalinity needs for nitrification and ethanol as the organic carbon dosing for denitrification. The ethanol carbon dosing requirements were estimated according to Ref. [8] and validated with the method from Ref. [29]. The kinetics for denitrification via ethanol were taken from Ref. [8]. The designed SBR had a tank depth of 6 m.

The thermal ammonia stripping and ammonia scrubber (STRIP) was designed based on [8,30]; and [31] (Fig. 2c). A mass-transfer rate for ammonium from the liquid to the gas of 0.0125 s^−1^ was assumed [8]. The henry constant for STRIP was 0.75 atm [8]. The thermal ammonia stripping and scrubber columns used 50 mm stainless steel pall rings with a packing factor of 107 m^2^ m^−3^. A stripping factor of 3 and a pressure drop of 400 N m^−1^ were assumed based on [8]. For the scrubber, a gas:liquid velocity difference of 75% was assumed [30].

All designs were evaluated using mass and energy balances and cross-checked with references, where possible. Additionally, it was assumed that any recovered energy or electricity from the WWTP (e.g., biogas or heat from combined heat power engines) was either exported or used elsewhere on site. Furthermore, all energy required for heating was included in the energy with the electricity. The stripping also assumed that the final product, such as ammonium hydroxide, was disposed of off-site via incineration.

### 2.3 Cost estimation

The equipment units designed for each technology in Fig. 2 were costed according to the cost curves presented in equation (1) and Table 2 [33]:

(1) $$C_{equipment}=a+b\times S^{n}$$

where *C* is the cost of the equipment, the intercept with the y-axis of the cost curve, *b* is the slope of the curve, *S* is the size parameter, and *n* is the exponent for the cost type in equation (1) [33].

**Table 2:** Cost curves for the main equipment and process units [32,33].

Item	Unit	S	S_upper_	H_igher_	a	b	n
Process tank	£ m^−3^	volume	10	4000	5800	1600	0.7
Heat exchanger	£ m^−2^	area	10	1000	28,000	54	1.2
Pump	£ L^−1^ s	flowrate	0.2	126	8000	240	0.9
Blower	£ m^−3^ h	flowrate	200	5000	4450	57	0.8
Mixer	£ kW^−1^	power	5	75	17,000	1130	1.05
Hydrocyclone	£ m^−3^	volume	0.1	1	0	35,000	1
Pall rings 50 mm	£ m^−3^	Volume			0	5000	1

The capital expenditure (CAPEX) for each technology was calculated based on the factorial method as the sum of all purchased equipment units multiplied by the Lang factor of 4.74 for fluid processing plants [33]. All prices for consumables and other items are presented in Table 3. The operational expenditure (OPEX) was calculated as the sum of energy, chemical, labour, material, heating, and other costs (e.g., disposal, freshwater, etc.). The OPEX was always presented on a per annum basis (pa) if not stated otherwise.

**Table 3:** Unit costs for the major consumables and items.

Item	Unit	Unit cost	Reference/note
Electricity	£ kWh^−1^	0.11	[34]
Fuel oil	£ t^−1^	65	[32]
Water	£ t^−1^	0.6	[32]
NaOH	£ t^−1^	469	[33]
Ethanol	£ t^−1^	600	[33]
Labour	£ h^−1^	20	[35]
Waste disposal via incineration	£ m^−3^	1.5	[33]

Any costs obtained in currencies other than British Pound (£) were converted with the currency exchange rates in the year the cost was obtained. Historical cost data were updated to the present date using equation (2) using the average inflation from the retail prices index [36].

(2) $$Cost\;in\;year\;A=Cost\;in\;year\;B\times {\left(1+Average\;inflation\right)}^{Year\;A-Year\;B}$$

The cost data were obtained on a US Gulf Coast basis (USGC) and were converted with location factors (LF) to a UK basis using equation (3). The effect of currency exchange and time have a strong impact on cost and were updated as described by Ref. [37]. All obtained costs were cross-checked where possible with different resources.

(3) $$Cost\;in\;location\;A=Cost\;in\;USGC\times LF_{A}$$

An asset lifespan of 40 years was assumed for each technology, and the whole-life cost (WLC) was estimated based on the method described by Ref. [37].

### 2.4 Evaluation and analysis

A sensitivity analysis was conducted on the cost of all technologies and scenarios. The parameters included in the sensitivity analysis were population growth, economic fluctuations, higher effluent qualities and environmental assumptions (Table 4). The population growth was reflected in higher sludge production and thus 20% greater influent load dewatering liquor load. The economic fluctuations were changes in cost and consumables, as shown in Table 4, which was based on consumer price indices and economic trends from Refs. [38,39]. The higher effluent qualities category is related to 50% lower ammonia, nitrite and nitrate concentration to further reduce the returned load to the mainstream wastewater treatment process. The environmental assumptions included changes to design that are usually defined based on on-site experiments or depend on the operation of the technology. The impact of these parameters was compared against the base-design case for the different technologies. The parameters were then grouped based on their impact on CAPEX and OPEX (see Table 5).

**Table 4:** Sensitivity analysis categories with the parameters changed and the percent change from the design value.

Category	Parameter changed	Percent change from design
Population growth	Increase in ammonia load	+20%
Higher effluent quality	Decrease in effluent ammonia concentration	−50%
	Decrease in effluent nitrite and nitrate concentration	−50%
Environmental assumptions	Fluctuation of MLSS concentration	±20%
	Fluctuation of the stripping factor	±20%
	Fluctuation in feed dewatering liquor temperature	+20%
	Reduction in ammonia to alkalinity ratio in the feed	−30%
	Fluctuation of anoxic and aerobic phases	±30%
Economic fluctuations	Equipment price change (e.g., pall rings)	±40%
	Increase in energy price	+30%
	Fluctuation in waste disposal cost	±50%
	Fluctuation operator wage	±30%

**Table 5:** Assumed and calculated characteristics for the three technologies (PNA, SBR and STRIP) for the two scenarios.

Characteristics	Assumed/Calculated	Scenario 1: THP/AD dewatering liquors			Scenario 2: conventional AD dewatering liquors		
		PNA^c^	SBR^d^	STRIP^e^	PNA^c^	SBR^d^	STRIP^e^
Feed flowrate, m^3^ d^−1^	Literature values^a^	1000	1000	1000	1000	1000	1000
Ammonia loading rate, kg N d^−1^	Literature values^a^	1700	1700	1700	750	750	750
BOD loading rate, kg d^−1^	Literature values^a^	450	450	450	203	203	203
TSS loading rate, kg d^−1^	Literature values^a^	500	500	500	500	500	500
Ammonia discharge limit, mg N L^−1^	Literature values^a^	70	70	70	70	70	70
TSS discharge limit, mg L^−1^	Literature values^a^	50	50	50	50	50	50
Nitrate discharge limit, mg N L^−1^	Literature values^b^	200	200	200	200	200	200
Reactor volume, m^3^	Calculated	1553	1250	46	1090	1250	35
Hydraulic retention time, h	Calculated	37.3	30.0	1.0	26.2	30.0	0.8
Reactor temperature, °C	Calculated	26	26	90	26	26	90
MLSS concentration, mg L^−1^	Calculated	3000	6150	NA	3000	6150	NA
MLVSS concentration, mg L^−1^	Calculated	1266	1281	NA	884	1281	NA
Dissolved oxygen set-point, mg L^−1^	Literature values^a^	0.3	2.0	NA	0.3	2.0	NA
Air flowrate, m^3^ min^−1^	Calculated	345	939	84	145	401	79
Alkalinity need as NaOH, kg d^−1^	Calculated	868	2540	0	0	0	0
Carbon need as ethanol, kg d^−1^	Calculated	0	4736	0	0	1688	0
Wash water requirement, kg d^−1^	Calculated	0	0	1330	0	0	1357
Mixing energy requirement, kW	Calculated	7.8	6.3	0	5.4	6.3	0
Energy requirement nitrogen removed, kW per kg N	Calculated	3	8	65	3	9	156

a Ochs et al. [43], Ochs et al. [27].

b Tchobanoglous et al. (2014).

c PNA: partial nitritation continuous stirred tank reactor with suspended sludge biomass.

d SBR: Nitrification/denitrification sequencing batch reactor.

e Thermal driven ammonia stripping with ammonia scrubber.

## 3 Results and discussion

### 3.1 Process design, mass and energy balances

All three technologies designed, and cost were able to reach the desired NH_4_–N 70 mg N L^−1^ discharge consent for both Scenarios 1 and 2 (Table 4). The PNA had a nitrogen loading rate (NLR) of 0.7–1.1 kg N m^−3^ d^−1^ and displayed ammonia removal efficiencies (ARE) of 90–95% (Table 4) for both Scenarios. Similar deammonification technologies with hydrocyclones operated with NLR of 0.5 kg N m^−3^ d^−1^ reached an ARE of 90% [11,40]. Based on the process design, the PNA required an airflow rate of 345 m^3^ min^−1^ for Scenario 1 and 145 m^3^ min^−1^ for Scenario 2 (Table 4). The different airflow rates related to the higher N–NH_4_ load of 1700 kg N d^−1^ for Scenario 1 (Table 4). The higher NH_4_–N load associated with THP dewatering was also the main reason for the higher reactor volume of 1553 m^3^ compared to 1090 m^3^ for Scenario 2. Based on an alkalinity mass balance, it was determined Scenario 1 required 868 kg d^−1^ of NaOH to raise the CO_2_/pH balance (Table 4.), while for Scenario 2, no NaOH dosing was required. Sodium hydroxide is commonly dosed to raise the pH, shift the CO_2_ balance, and make more alkalinity available.

The SBR was designed for an NLR of 0.6–1.4 kg N m^−3^ d^−1^ achieving an ARE of >95% for Scenario 1 and Scenario 2. Conventional nitrification/denitrification operates at NLR of 0.2–0.4 kg N m^−3^ d^−1^ with ARE of 95–99% [41]. The SBR had a reactor volume of 1250 m^3^, and the MLSS was 6150 mg L^−1^ for both Scenarios (Table 4). The air flowrate in the SBR was 939 and 401 m^3^ min^−1^ for Scenario 1 and Scenario 2, respectively. Based on an alkalinity balance, it was determined that only Scenario 1 required an alkalinity dosing of 2540 kg d^−1^ of NaOH (Table 4). Additionally, carbon addition was also required for denitrification due to the low BOD concentration of 203–405 mg L^−1^ (Table 1) in the influent dewatering liquors. The SBR required 4736 and 1688 kg d^−1^ of ethanol (external carbon source) for Scenarios 1 and 2, respectively (Table 4). Previous studies reported that nitrification/denitrification sidestream technologies require organic carbon supplementation to reach effluent limits of <200 mg N L^−1^ [24].

The STRIP was designed for an NLR of 21.7–36.7 kg N m^−3^ d^−1^, achieving an ARE of 95%, meeting the design discharge limits. Based on Henry's law and thermodynamic principles, the optimum temperature for the stripping was 90 °C (Table 4) [8]. This meant that STRIP required 3114 kW d^−1^ for heating. Furthermore, the stripping column had an air-flowrate of 79–84 m^3^ min^−1^ (Table 4). Furthermore, the scrubber produced 1330–1357 kg d^−1^ of ammonium hydroxide (1–2% weight weight^−1^) (Table 4). The STRIP had a volume of 45 m^3^ for Scenario 1 and 35 m^3^ for Scenario 2. The stripping column was 69% of the total STRIP volume, and the scrubbing column was 31%. No chemical addition was required for the STRIP.

When comparing the designs of the different technologies, the PNA displayed a 67% lower air requirement than the SBR. Additionally, the PNA did not require any chemical addition for Scenario 2, but alkalinity dosing was needed for Scenario 1. However, the PNA required 90% fewer chemicals than the SBR. This aligns with previous literature where deammonification technologies had a 60% lower oxygen requirement than nitrification/denitrification [9,42].

### 3.2 Economic evaluation

**Fig. 3:**
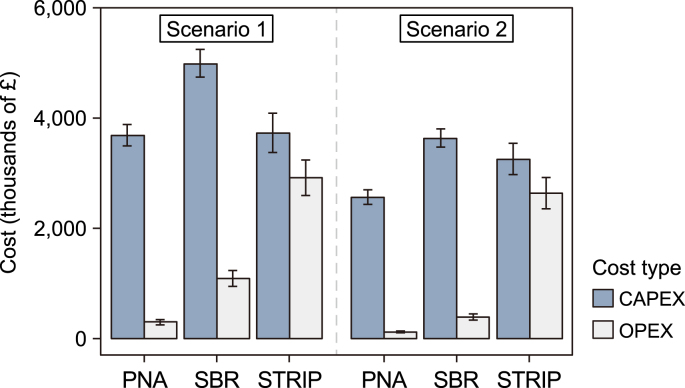
Capital expenditure (CAPEX) and operational expenditure (OPEX) for three technologies: deammonification (PNA), nitrification/denitrification sequencing batch reactor (SBR) for Scenarios 1 and 2. The error bars indicate the standard deviation from the sensitivity analysis on the major design assumptions.

**Fig. 4:**
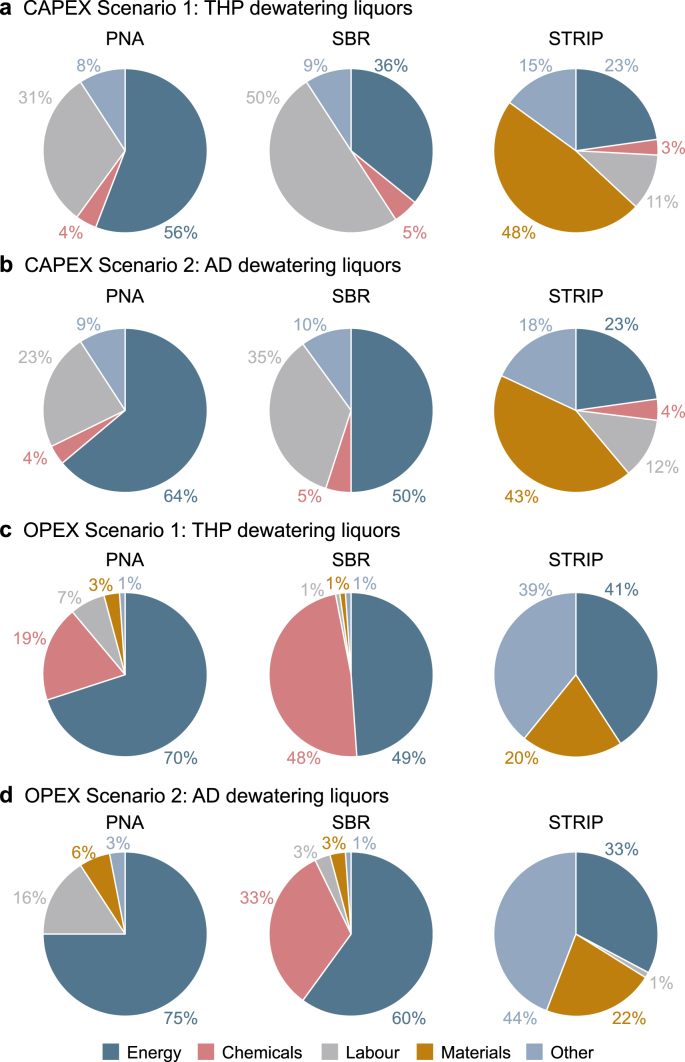
**a**–**b**, Breakdown of capital expenditures (CAPEX) for Scenario 1 (**a**) and Scenario 2 (**b**) for the three-tested technologies. The CAPEX for Scenario 1 was PNA: £3,689k, SBR: £4,994k, and STRIP: £3,734k. The CAPEX for Scenario 2 was PNA: £2,565k, SBR: £3,637k, and STRIP: £3,255k. **c–d**, Breakdown of operational expenditure (OPEX) for Scenario 1 (**c**) and Scenario 2 (**d**) for the three tested technologies. The OPEX for Scenario 1 was PNA: £294k pa, SBR: £1,093k pa, and STRIP: £2920 pa. The OPEX for Scenario 2 was PNA: £119k, SBR: £390k, and SRTP: £2,640k.

The three sidestream technologies designed varied greatly in CAPEX and OPEX. For Scenario 1, a CAPEX of £3,689k, £4,994k, and £3,734k was estimated for PNA, SBR and STRIP, respectively (Fig. 3). For Scenario 2, the CAPEX values were lower at £2,565k, £3,637k, and £3,255k for PNA, SBR and STRIP respectively. The most expensive equipment for the PNA and SBR was the biological reaction tanks, whereas the most expensive equipment for the STRIP was the packaging material (Fig. 4). Another high cost, representing 20–50% of the CAPEX, was the air blowers. Additional process units included the hydrocyclone, chemical dosing or other required process units related to 10–15% of the CAPEX. Previous studies also reported that one of the main CAPEX of biological nitrogen removal technologies was the tanks with 35–60% [37,44].

The OPEX for Scenario 1 was £294k pa, £1093k pa, and £2920 pa for PNA, SBR and STRIP, respectively (Fig. 4). Whereas the OPEX for Scenario 2 was £119k, £390k, and £2640k for respective three technologies. The OPEX can be broadly split into five major groups: energy, chemicals, labour, materials and other (e.g., waste disposal, freshwater, etc.). The main contributor for OPEX for SBR, PNA and STRIP was the energy cost of 49–60% and 70–75% and 33–41%, respectively (Fig. 4). The PNA required 37% less energy as well as 90–100% less chemical dosing than the SBR. Previous studies demonstrated that deammonification technologies are more cost-effective than nitrification/denitrification technologies [23,24], due to savings in energy for aeration and no need for chemical dosing [23,24]. No peer-reviewed studies were found that evaluate the cost of air stripping or thermal ammonia stripping for the sidestream application at wastewater treatment plants. It was demonstrated that the PNA and STRIP presented 25–28% and 15–28% lower CAPEX compared to the SBR for both scenarios. The PNA also presented 64–71% lower OPEX than the SBR for both scenarios, whereas the STRIP presented up to nine times higher OPEX than the PNA.

**Fig. 5:**
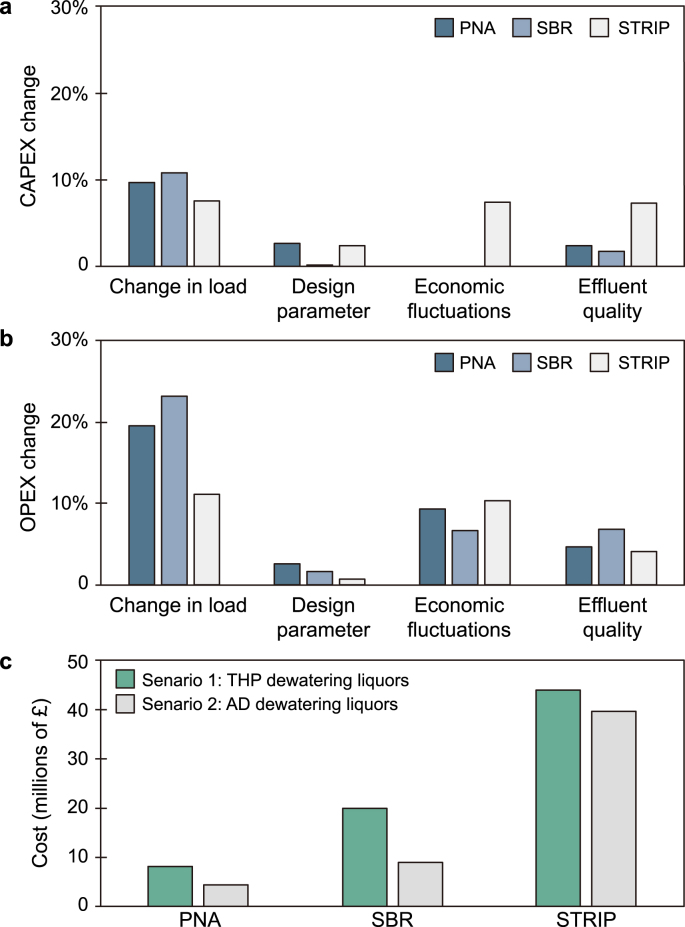
**a**, Results of the sensitivity analysis for CAPEX as the percentage change from the base case for change in load, design parameter, economic fluctuation and effluent quality for CAPEX and OPEX. **b**, Results of the sensitivity analysis for OPEX as the percentage change from the base case for change in load, design parameter, economic fluctuation and effluent quality for CAPEX and OPEX. **c**, Whole-life cost of the three tested technologies (PNA, SBR, and STRIP) for the two dewatering liquor streams (Scenarios 1 and 2).

Overall, the highest whole-life cost was estimated for the STRIP technology for both scenarios at £39.7M–£43.9 M (Fig. 5). The WLC of the SBR was half of the STRP for Scenario 1 (£20.1 M) and only £9.0 M for Scenario 2. The PNA obtained the lowest WLC, displaying £7.7 M and £4.2 M for Scenarios 1 and 2, respectively (Fig. 5).

### 3.3 Sensitivity analysis

The sensitivity analysis revealed that ammonia load had the highest impact on the variation in both CAPEX (9%) and OPEX (15%) for PNA, SBR, and STRIP. The OPEX of the SBR was most impacted by population growth and higher effluent quality, with 21% and 7%, respectively. Large price variations of the packaging material in the STRIP impacted the CAPEX by 7%. The price of the packing material for stripping columns may vary between £1000 m^−3^ to £8300 m^−3^ [32,33]. The highest impact on OPEX for STRIP was, in equal proportion, the waste disposal cost for the disposal of ammonium hydroxide and the energy cost (Fig. 3). The higher load with the THP/AD dewatering liquors, in Scenario 1, had the highest impact on the SBR where the whole-life cost doubled compared to Scenario 2. The STRIP had the lowest cost fluctuation between Scenario 1 of £43,939k and Scenario 2 of £39,655k. Past literature widely discussed the benefits of THP/AD dewatering liquors, but no costs have been associated to date with the additional ammonia concentrations. This study showed that the higher ammonia load in THP/AD dewatering liquors led to a 2-fold increase in the whole-life cost.

### 3.4 Ammonia recovery and climate change mitigation

The investment cost based on nitrogen removed was for the PNA £6.3–10.4 kg^−1^ N, £8.4–14.7 kg^−1^ N, and for the STRIP £6.0–12.4k kg^−1^ N for Scenarios 1 and 2, respectively. Another study reported that the investment costs £1.6 kg^−1^ N for both deammonification technology and nitrification/denitrification [23]. Whereas [24] reported £23.4 kg^−1^ N investment was required for a deammonification reactor. The operational cost normalised by nitrogen removed were for the PNA £0.2–0.5 kg^−1^ N, for the SBR £0.6–1.8 kg^−1^ N and the STRIP £4.4–4.8 kg^−1^ N for Scenarios 2 and 1, respectively. This was lower than reported by other studies reporting £1.4–5.1 kg^−1^ N for deammonification and £1.6–5.3 kg^−1^ N for nitrification/denitrification [23,24]. Based on the cost data, the deammonification technologies were best suited for application for both scenarios.

However, two key characteristics should also be considered when selecting a new sidestream nitrogen removal technology. The first is climate change mitigation by reducing nitrous oxide emissions, and the second is the goals of the water industry to contribute to the circular economy goals and promote nitrogen recovery. Over recent years an increasing number of researchers and utilities have recognised the need for nitrogen recovery and deliver a water industry circular economy [45,46]. The STRIP recovered around 1350 kg d^−1^ of low-strength ammonium hydroxide solution with a concentration of 1% weight weight^−1^. The potential recovered product of ammonium hydroxide has various applications, including household cleaners, food production, and avoiding nitrogen abstraction from the atmosphere via the Haber-Bosch process. The ammonium hydroxide concentration can be further increased via fractionating or even further processed to other ammonia salts, such as ammonia sulphate, by combining it with sulphuric acid to produce [21]. Previous studies on thermal ammonia stripping were conducted on a pilot-scale and aimed to recover the potential of ammonia sulphate but did not quantify the final products [17,18]. In general, much of the past research focused on ammonia stripping as a recovery process evolved around the process parameters [21,22] and only a few studies focused on nitrogen recovery via fertiliser [21]. The STRIP could overall recover 49 kg N pa. Past studies focused on ammonia recovery with other nitrogen recovery technologies reported a nitrogen recovery of 90–120 kg N pa [37,47]. However, it is understood that much of the recovery potential of the products depends on market demand, supply chain, product purity, and quantity [22,48]. Another important consideration for nitrogen recovery evolves around the recovered product policies and does not proactively promote the circular economy [20,22].

It is well understood that biological process emits a range of greenhouse gasses from the oxidation of organic matter and nitrous oxide from nitrogen removal [19]. Ammonia removal with PNA and SBR impacts the environment due to nitrous oxide emissions, whereas no nitrous oxide is emitted with STRIP. Different process parameters have been associated with higher nitrous oxide emission of deammonification and nitrification/denitrification technologies, including the operation at low oxygen concentration, nitrite accumulation and low C/N ratios of the wastewater [49]. Chemical addition in the SBR could further lead to elevated carbon dioxide emissions from the bacterial reactions [8]. In a comparison between deammonification technologies and conventional nitrification/denitrification, it was found that the first emitted less nitrous oxide [50,51]. However, past studies also highlighted that the nitrous oxide emissions of deammonification can exceed the nitrification/denitrification when leaving the partial nitritation step uncontrolled with nitrite concentrations >20 mg N L^−1^ [52,53]. Additionally, a range of indirect greenhouse gas emissions are associated with the power consumption from the grid. The STRIP and the SBR have the overall highest energy requirement of 65–156 and 8–9 kW kg^−1^ N, respectively, which could contribute to a higher carbon footprint, while deammonification had the lowest energy requirement of 3 kW kg^−1^ N. Past research often focused on the energy optimisation of biological processes and thus only tackled indirect greenhouse gas emissions [22].

## 4 Conclusions

In this study, three different sidestream ammonia removal technologies (i.e., PNA, SBR, and STRIP) were designed and compared for WLC in two scenarios, conventional AD and THP/AD dewatering liquors.

• PNA demonstrated the overall lowest CAPEX, OPEX, with a whole-life cost of £7.7 M.
• The treatment of THP dewatering liquors leads to an increase in whole-life cost between 1 and 3 times the cost compared to AD dewatering liquors.
• Ammonia load and the energy price caused the biggest impact on the cost of biological technologies (PNA and SBR). Packing material costs and energy prices had the biggest impact on STRIP.
• For both scenarios, PNA also demonstrated the lowest cost for eliminating nitrogen with £0.2–0.5 kg^−1^ N.
• STRIP has the potential for nitrogen recovery of 49 kg N pa.
• Technology selection should include multivariable characteristics, including greenhouse gas emissions, recovery potential, treatment capability, and whole-life cost.

## Declaration of competing interest

The authors declare that they have no known competing financial interests or personal relationships that could have appeared to influence the work reported in this paper.
